# Nitroglycerin challenge identifies microcirculatory target for improved resuscitation in patients with circulatory shock

**DOI:** 10.1186/s40635-024-00662-3

**Published:** 2024-09-02

**Authors:** Massimiliano Bertacchi, Pedro D. Wendel-Garcia, Anisa Hana, Can Ince, Marco Maggiorini, Matthias P. Hilty

**Affiliations:** 1 Institute of Intensive Care Medicine, University Hospital of Zurich, Zurich, Switzerland; 2 Laboratory of Translational Intensive Care, Department of Intensive Care, Erasmus MC, University Medical Center, Rotterdam, The Netherlands

**Keywords:** Microcirculation, Critical care, Sublingual microcirculatory assessment, Microcirculatory reserve capacity, Capillary recruitment, Resuscitation

## Abstract

**Background:**

Circulatory shock and multi-organ failure remain major contributors to morbidity and mortality in critically ill patients and are associated with insufficient oxygen availability in the tissue. Intrinsic mechanisms to improve tissue perfusion, such as up-regulation of functional capillary density (FCD) and red blood cell velocity (RBCv), have been identified as maneuvers to improve oxygen extraction by the tissues; however, their role in circulatory shock and potential use as resuscitation targets remains unknown. To fill this gap, we examined the baseline and maximum recruitable FCD and RBCv in response to a topical nitroglycerin stimulus (FCD_NG_, RBCv_NG_) in patients with and without circulatory shock to test whether this may be a method to identify the presence and magnitude of a microcirculatory reserve capacity important for identifying a resuscitation target.

**Methods:**

Sublingual handheld vital microscopy was performed after initial resuscitation in mechanically ventilated patients consecutively admitted to a tertiary medical ICU. FCD and RBCv were quantified using an automated computer vision algorithm (MicroTools). Patients with circulatory shock were retrospectively identified via standardized hemodynamic and clinical criteria and compared to patients without circulatory shock.

**Results:**

54 patients (57 ± 14y, BMI 26.3 ± 4.9 kg/m^2^, SAPS 56 ± 19, 65% male) were included, 13 of whom presented with circulatory shock. Both groups had similar cardiac index, mean arterial pressure, RBCv, and RBCv_NG_. Heart rate (*p* < 0.001), central venous pressure (*p* = 0.02), lactate (*p* < 0.001), capillary refill time (*p* < 0.01), and Mottling score (*p* < 0.001) were higher in circulatory shock after initial resuscitation, while FCD and FCD_NG_ were 10% lower (16.9 ± 4.2 and 18.9 ± 3.2, *p* < 0.01; 19.3 ± 3.1 and 21.3 ± 2.9, *p* = 0.03). Nitroglycerin response was similar in both groups, and circulatory shock patients reached FCD_NG_ similar to baseline FCD found in patients without shock.

**Conclusion:**

Critically ill patients suffering from circulatory shock were found to present with a lower sublingual FCD. The preserved nitroglycerin response suggests a dysfunction of intrinsic regulation mechanisms to increase the microcirculatory oxygen extraction capacity associated with circulatory shock and identifies a potential resuscitation target. These differences in microcirculatory hemodynamic function between patients with and without circulatory shock were not reflected in blood pressure or cardiac index.

**Supplementary Information:**

The online version contains supplementary material available at 10.1186/s40635-024-00662-3.

## Background

Circulatory shock and multi-organ failure remain primary contributors to morbidity and mortality in critically ill patients. Circulatory shock, defined as a life-threatening state of insufficient tissue perfusion and oxygenation, is closely related to hemodynamic impairment on a macro- and microcirculatory level [1], and it is generally accepted that the goal of resuscitation should be the restoration of tissue perfusion and oxygenation. It remains challenging, however, to use the microcirculatory function as a resuscitation target, not only because of a lack of techniques incorporated in resuscitation procedures targeting tissue perfusion, but foremost because of a lack of understanding of the intrinsic regulation mechanisms of the microcirculation in response to reduced oxygen availability in the tissues and their role in circulatory shock, which is elemental in the definition of treatment targets. Previously, a clear association has been shown between the degree of sublingual microcirculatory dysfunction and outcome in adults [2–4] and children [5] presenting with circulatory shock, with a reduced functional capillary density (FCD), capillary red blood cell flow velocity (RBCv), or inter-capillary heterogeneity representing the most frequently observed abnormalities [6–9]. To counteract reduced oxygen availability in the tissue, as examinations in hypoxemic healthy volunteers exposed to high altitude have suggested [10, 11], regulation mechanisms intrinsic to microcirculation, such as the recruitment of FCD, may provide for an increase in oxygen extraction capacity in the tissue by decreasing diffusion distances, independent of cardiac output and arterial blood pressure. Previous observations in patients with circulatory shock have shown that the topical sublingual application of acetylcholine further increased FCD after systemic administration of dobutamine in patients with septic shock [12], and in comparison to native measurements in patients with cardiogenic shock [6], revealing the presence of microcirculatory reserve capacity which can be utilized for improving oxygen extraction and thereby optimizing resuscitation. However, acetylcholine-dependent vasodilation relies on proper endothelial cell function which may be altered in conditions of inflammation, thus not revealing the full reserve capacity. An endothelial cell-independent alternative may be the use of a topical excess of nitroglycerin. Inconsistency between the extent of intrinsic compensation and the nitroglycerin response could also help explain the loss of coherence between the macro- and microcirculation that has previously been described in these patients [7, 9]. To explore the characteristics of the nitroglycerin-elicited microcirculatory reserve capacity in patients being resuscitated from circulatory shock, in the present study we compared the native sublingual microcirculatory FCD and capillary RBCv to the changes in these variables as a result of topical nitroglycerin administration sublingually in patients admitted to the ICU with circulatory shock after the initial resuscitation and those without circulatory shock. Our hypothesis was that (I) patients suffering from circulatory shock have a lower native FCD and RBCv as compared to patients without circulatory shock, and (II) they present with a lower microcirculatory reserve capacity as identified by a nitroglycerin challenge as compared to patients without circulatory shock as a sign of intrinsic compensatory mechanisms of the microcirculation to increase oxygen extraction in the tissue. We propose that sublingual handheld vital microscopy offers a technique to quantitatively measure FCD and RBCv as the determinants of tissue perfusion and that a topical nitroglycerin challenge would allow identification of recruitable microcirculatory fraction still in need of resuscitation.

## Methods

In this prospective observational study, mechanically ventilated patients admitted to the ICU of the University Hospital of Zurich from January 1st, 2018, to January 31st, 2019 were consecutively screened for inclusion. Patient inclusion in the study was concluded when the intended number of patients presenting with circulatory shock was reached. The study was approved by the ethics committee of the Canton of Zurich (BASEC 2017-01564, ClinicalTrials.gov Identifier: NCT03651635) and conducted in accordance with the Declaration of Helsinki.

### Study design

Inclusion criteria were mechanical ventilation and the availability of research personnel to perform microcirculatory measurements in the first 24 h after admission and after the initial resuscitation according to the clinical standard of practice [13]. Exclusion criteria were age under 18 years and missing explicit consent of the patient or legal representative. Written informed consent for participation from the patient, or in case of death or disability, from the next of kin or legal representative was sought for every included patient. Patients were then retrospectively divided into two groups. Patients with persistent signs of circulatory shock after initial resuscitation, as defined by the presence of more than three of the following criteria: cardiac index < 2.2 l/min/m^2^ [1], lactate concentration > 2 mmol/l [14], vasopressor dependency index > 3 [15], Mottling score ≥ 2 [16], capillary refill time > 3 s [17], mean arterial pressure < 65 mmHg [14], or the need for veno-arterial extracorporeal membrane oxygenation [18], were assigned to the circulatory shock group. All criteria for circulatory shock were recorded at the time of microcirculatory measurement. The other patients were assigned to the control group. The hypothesis was tested by comparing the sublingual microcirculatory hemodynamics between the two groups, with the difference in FCD serving as the primary end point. As a secondary end point, we analyzed RBCv, the nitroglycerin response, and the correlation between micro- and the macrocirculatory variables. Finally, we analyzed the differences in the microcirculatory variables in survivors and non-survivors at 28 days.

### Measurement of the sublingual microcirculation and clinical variables

**Fig. 1:**
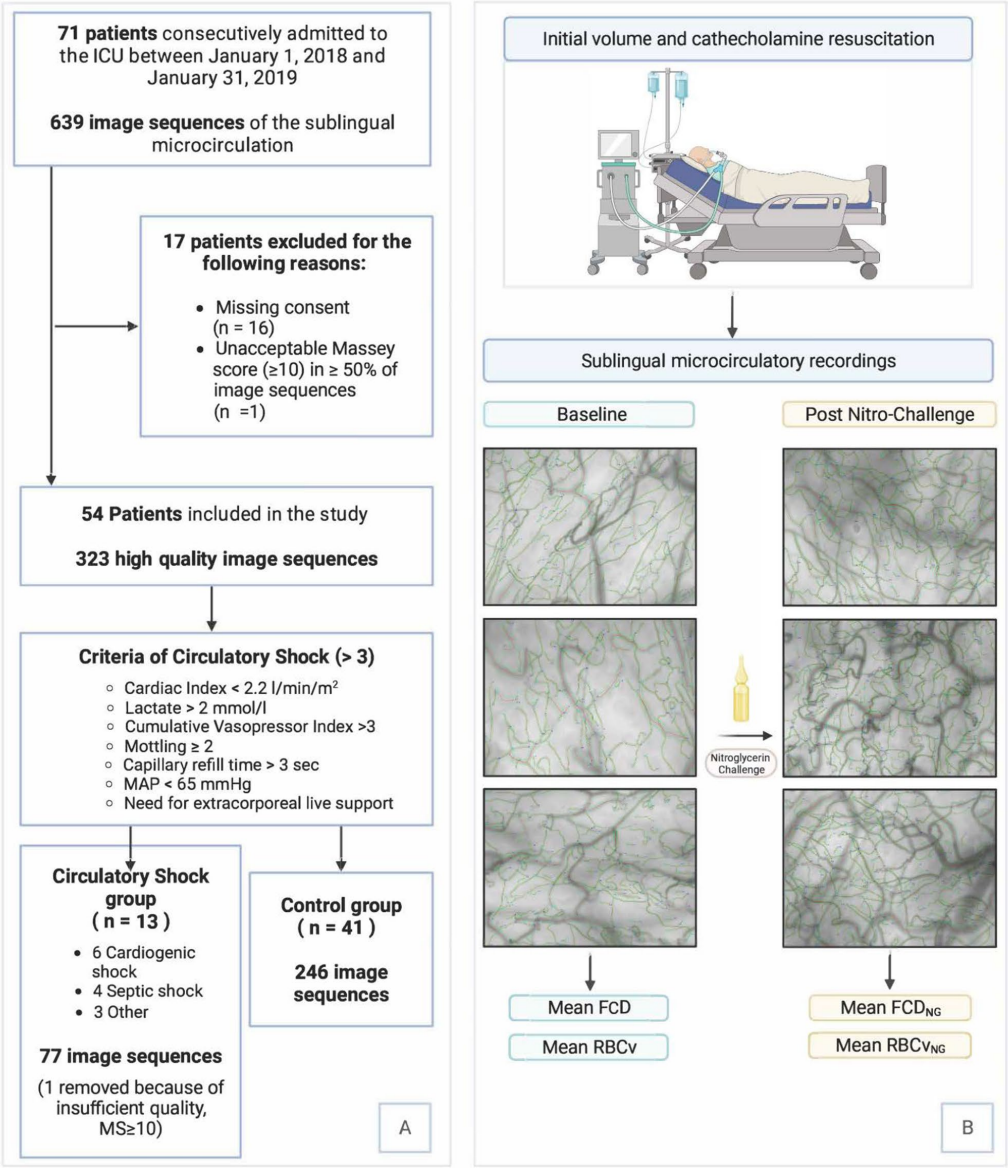
Study flowchart, including the number of microcirculatory measurements for each group (**A**) and representation of the study design with the initial cardio-respiratory resuscitation at admission, followed by the microcirculatory measurements of sublingual capillaries. The left column with the baseline measurement in three territories, with the extraction of FCD and RBCv, and the right column with the measurements of three territories after the nitroglycerin challenge with the extraction of FCD_NG_ and RBCv_NG_ (**B**). *MS* Massey score, *MAP* mean arterial pressure, *FCD* functional capillary density, *RBCv* red blood cell velocity, *FCD*_*NG*_ maximal recruitable functional capillary density, *RBCv*_*NG*_ maximal recruitable red blood cell velocity

The sublingual microcirculation was assessed by performing a bedside optical visualization of sublingual capillary bed with incident dark-field imaging [19, 20] by gently placing a handheld vital microscope under the tongue. Sublingual microcirculation was measured within the first 24 h after admission, after the initial volume and catecholamine resuscitation as performed by independent physicians in the ICU according to the clinical standard of practice. Following the current guidelines [21] and a recently described method to assess recruitability using the topical application of nitroglycerin [22], six recordings of sublingual microcirculation were performed for each measurement using a high-resolution incident dark-field handheld vital microscope (Braedius Medical, Huizen, The Netherlands) placed without pressure on the sublingual mucosa. Three initial recordings of 20 s (250 frames) were taken in different sublingual territories, and three additional recordings 60 s after the topical administration of three drops (150 μl) of 1% nitroglycerin (4.4 × 10^−2^ M, diluted in 1:100 NaCl 0.9%) for a final dose of 0.015 mg per application. This protocol has previously been demonstrated to avoid impact on systemic blood pressure and leave no measurable traces of nitroglycerin metabolites in the systemic circulation [23]. All image sequences of the sublingual microcirculation were recorded and stabilized using the CCTools software [24] (Braedius Medical, Huizen, The Netherlands) and assessed using the Massey score [25]. Recordings with a Massey score ≥ 10 were excluded from further analysis. Acceptable quality in > 50% of all image sequences per patient was required for analysis. Automated image sequence analysis and calculation of FCD and capillary RBCv, considering all vessels with a diameter < 20 µm, was then performed using the MicroTools advanced computer vision software (Active Medical BV, Leiden, The Netherlands) [26]. The mean of the three initial measurements was reported as the native microcirculatory FCD and RBCv, and the mean of the three measurements after application of a topical sublingual nitroglycerin challenge was reported as FCD_NG_ and RBCv_NG_ (Fig. 1B). The difference between the two measurements was reported as ΔFCD_NG_ and ΔRBCv_NG_. Arterial blood pressure and central venous pressure were measured immediately before each microcirculatory measurement, via a fluid-filled catheter inserted into the radial artery and the internal jugular vein, respectively. Mottling score and capillary refill time were assessed by trained personnel at the same time as described in detail elsewhere [16, 17]. Cardiac output was measured either via transpulmonary thermodilution (PiCCO, Getinge, Gothenburg, Sweden) or continuous cardiac output monitoring via a Swan–Ganz catheter (Edwards Life Sciences, Irvine, USA). Arterial blood gas analysis, SAPS, and SOFA scores were extracted from the patient data monitoring system, and 28-day survival was recorded during follow-up.

### Statistical analysis

All variables were tested for normal distribution using QQ plots, confirming normal distribution of all variables except for the total fluid administration. Results are reported as mean ± SD for continuous, normally distributed variables, and median (IQR) for non-normally distributed variables. Comparisons of microcirculatory variables between both groups were made using linear mixed model analysis with circulatory shock status entered as fixed effects and intercepts for subjects and per-subject random slopes representing the effect on the dependent variables entered as random effects. Linear correlation was used to descriptively assess potential associations between microhemodynamic variables measured in the sublingual microcirculation, macrohemodynamic variables, and capillary refill time. Pearson’s product–moment correlation coefficient was calculated, and *r* ≥ 0.6 or *r* ≤ − 0.6 in the presence of a positive statistical significance test was considered as relevant. Bonferroni’s correction was applied to the *p* values resulting from linear correlation analysis of the individual variables (*n* = 48 tests). The relationship between microcirculatory variables and survival at 28 days was tested via Student’s *t* test. For sub-group analysis differentiating septic and cardiogenic shock from other forms of shock, microcirculatory variables were compared via one-way analysis of variance. The sample size needed was estimated based on previous data, with critically ill patients suffering from septic and cardiogenic shock presenting with a difference in FCD of − 3.7 ± 3.9 and − 6.7 ± 3.9 mm/mm^2^ as compared to a general ICU population [27]. Thus, to detect a difference in means of FCD induced by any form of circulatory shock versus in an ICU population, using a two-sample *t* test with an 80% power and 5% two-sided type I error rate, the inclusion of at least 13 patients in each group was targeted. Data analysis was performed with JMP (SAS Editor, version 15.2.1) and R (version 4.1.2). Figures were created with JMP and with a licensed version of biorender.com. A two-sided *p* value < 0.05 was considered statistically significant.

## Results

### Patient characteristics

Data from 71 patients were collected, of which 16 were excluded because of missing informed consent and 1 because of poor image sequence quality. A total of 54 patients were thus included in the study, with 323 recorded image sequences (Fig. 1A). Seven image sequences were excluded from analysis due to insufficient video quality (Massey score ≥ 10). Microcirculatory measurements were performed 10.3 ± 7.1 h after ICU admission. All included patients were mechanically ventilated and severely ill, with a mean admission SAPS score of 57.3 ± 18.2 and a mean SOFA score of 9.4 ± 4.0 (Table 1). 13 patients fulfilled the criteria for persistent signs of circulatory shock, and 41 were assigned to the control group. Among patients with circulatory shock, six patients presented with cardiogenic shock, four with septic shock, and three with other types of shock. In the control group, 22 patients were diagnosed with neurologic or respiratory disease, 8 patients with sepsis, and 11 patients with heart failure. Three patients with circulatory shock and four patients in the control group were treated with veno-arterial extracorporeal membrane oxygenation (see Supplementary Table S3 for microhemodynamic variables). Patients with circulatory shock presented with higher SAPS score (*p* = 0.03), heart rate (*p* < 0.001), lactate levels (*p* < 0.001), vasopressor dependency index (*p* < 0.001), Mottling score (*p* < 0.001), capillary refill time (*p* < 0.01), and central venous pressure (*p* = 0.02, see Table 1). Total fluid administration during the initial resuscitation phase amounted to 3850 (167–5380) ml in patients with circulatory shock and 1635 (987–2285) ml in the control group. Patients with cardiogenic shock received 1773 (650–2897) ml. Survival at 28 days was 54% in patients presenting with circulatory shock and 73% in the control group.

**Table 1:** Patient characteristics in the circulatory shock group and the control group

	All patients *n* = 54	Circulatory shock group *n* = 13	Control group *n* = 41	Between group *p* value
Patient characteristics				
Age [a]	56.9 ± 14.0	59.1 ± 13.6	54.2 ± 14.2	0.53
Body mass index [kg/m^2^]	26.1 ± 5.0	24.7 ± 4.5	26.6 ± 4.6	0.18
Sex (male)	35 (65%)	9 (69%)	26 (63%)	0.70*
SAPS score at ICU admission [1]	57 ± 18	68 ± 18	54 ± 17	**0.03**
SOFA score at ICU admission [1]	9 ± 4	11 ± 4	9 ± 4	0.15
Acute kidney injury	30 (6%)	9 (69%)	21 (51%)	0.25*
Chronic kidney disease	15 (28%)	4 (31%)	11 (28%)	0.82*
Extracorporeal support therapy				
VA-ECMO	7 (13%)	3 (20%)	4 (10%)	0.24*
Macrohemodynamic function variables				
Cardiac index [l/min/m^2^]	2.9 (2.0–4.2)	2.9 (1.9–4.6)	2.9 (2.5–3.7)	0.82
Temperature [°C]	36.70 ± 1.1	36.7 ± 1.8	35.8 ± 5.8	0.99
Systolic arterial pressure [mmHg]	103.5 (93.7–114)	101 (86–113)	107 (96–114)	0.13
Mean arterial pressure [mmHg]	70 (64–75)	65 (63–72)	71 (66–76)	0.06
Diastolic arterial pressure [mmHg]	52.5 (58.3–47.8)	54 (52–58)	53 (47–58)	0.59
Heart rate [s^−1^]	875 (73.5–102.8)	107 (93–113)	84 (69–92)	**< 0.001**
Central venous pressure [mmHg]	11.5 (9–15)	13 (10–17)	11 (9–13)	**0.02**
Vasopressor dependency index [1]	2.37 (0.6–5.6)	6.3 (5.5–9.0)	1.3 (0.3–3.3)	**< 0.001**
Laboratory analysis				
Lactate [mmol/l]	2.0 (1.2–4.2)	3.4 (3.0–7.8)	1.5 (1.0–3.0)	**< 0.001**
Hematocrit [1]	0.34 (0.30–0.40)	0.32 (0.29–0.34)	0.35 (0.28–0.38)	0.46
Hemoglobin [g/l]	110 (87–127)	102 (94–110)	112 (89–128)	0.67
Clinical signs of microcirculatory impairment				
Mottling score [1]	0 (0–1)	2 (0–2)	0 (0–0)	**< 0.001**
Capillary refill time [s]	4 (3–5)	5 (4–6)	4 (3–5)	**< 0.01**
Outcome				
28-day survival	37 (69%)	7 (54%)	29 (70%)	0.20*

*SAPS* Simplified Acute Physiology Score, *SOFA* Sequential Organ Failure Assessment, *ICU* intensive care unit, *VA-ECMO* veno-arterial extracorporeal membrane oxygenation

*P* values were calculated via a mixed-effects model of each variable with circulatory shock status entered into the model as fixed effect. For categorical variables, the *p* values were calculated via a Chi-square test of independence (indicated with *). P values < 0.05 are highlighted in bold. Results for categorical variables are reported as *n* (%), continuous variables as mean ± standard deviation, or median (IQR) as appropriate

### Native microcirculatory function in patients with and without circulatory sock

**Fig. 2:**
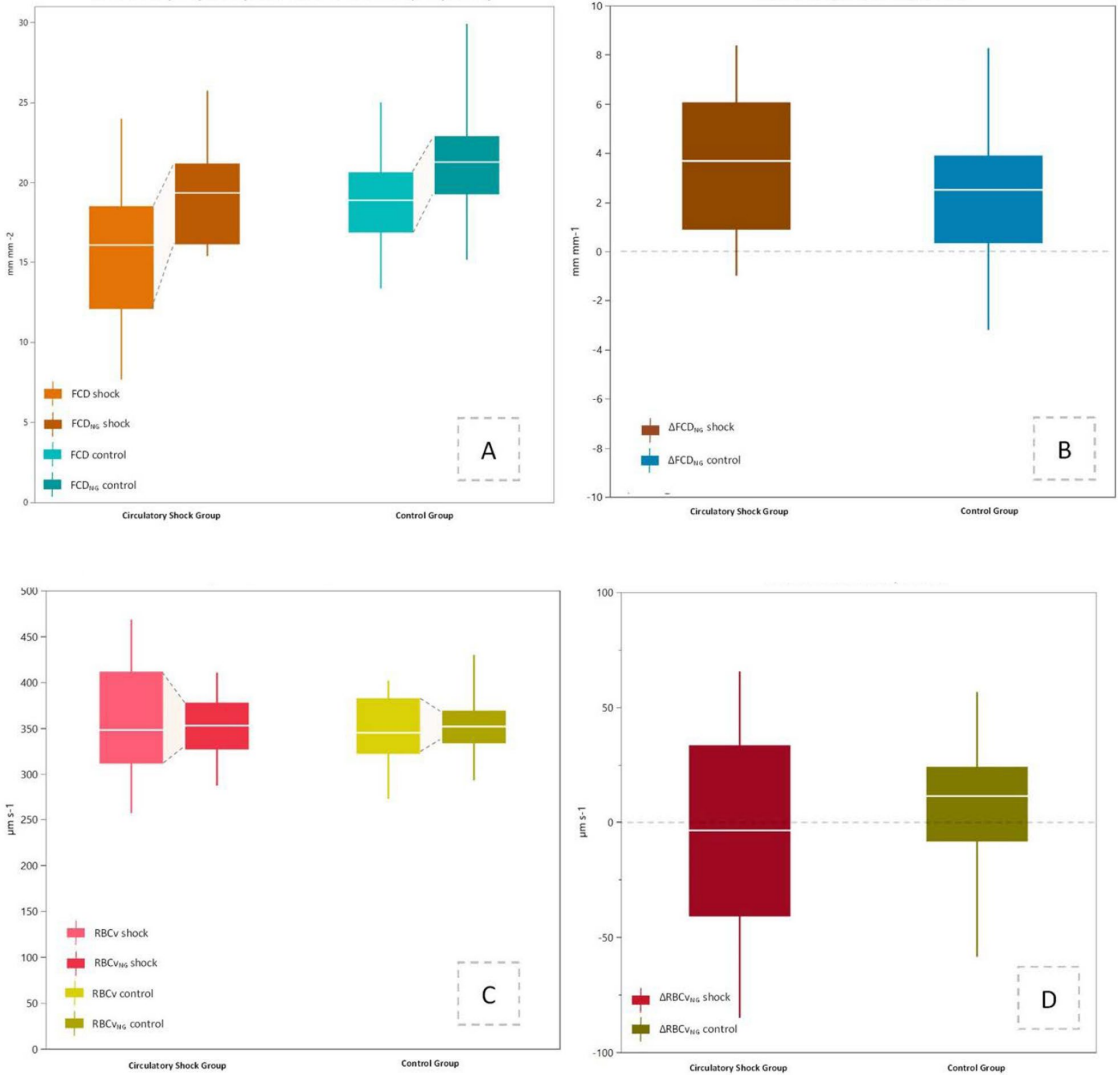
FCD and FCD_NG_ were lower in patients with circulatory shock as compared to the control group, while circulatory shock patients reached FCD_NG_ similar to FCD in patients without shock (**A**). Nitroglycerin response was similar in both groups (**B**, **D**). RBCv and RBCv_NG_ were similar in both groups (**C**). Boxplots represent median and IQR, the whiskers represent range, and the dashed gray lines in **B** and **D** represent the zero effect line for the nitroglycerin response. *FCD* functional capillary density, *RBCv* red blood cell velocity, *FCD*_*NG*_ maximal recruitable functional capillary density, *RBCv*_*NG*_ maximal recruitable red blood cell velocity, *ΔFCG*_*NG*_ FCD nitroglycerin response, *ΔRBCv*_*NG*_ RBCv nitroglycerin response

Patients with circulatory shock had a 15% lower FCD as compared to the control group (*p* < 0.01), while RBCv did not differ between the two groups (*p* = 0.48; Fig. 2A, Supplementary Table S1). 16 patients presented with elevated Mottling score [*n* = 8 (62%) and *n* = 8 (20%) in the circulatory shock and control groups, respectively] and 42 patients with prolonged capillary refill time (*n* = 11, 85% and *n* = 31, 76% in the circulatory shock and control groups, respectively; Table 1). No correlation was found between Mottling score and FCD, RBCv, or the nitroglycerin response in sublingual microcirculation (*p* > 0.05). Higher values of capillary refill time were associated with lower values of RBCv (*r* = − 0.82, *p* < 0.001) and higher ΔRBCv_NG_ (*r* = − 0.71, *p* = 0.01) in patients with circulatory shock.

### Nitroglycerin response in the sublingual microcirculation

**Fig. 3:**
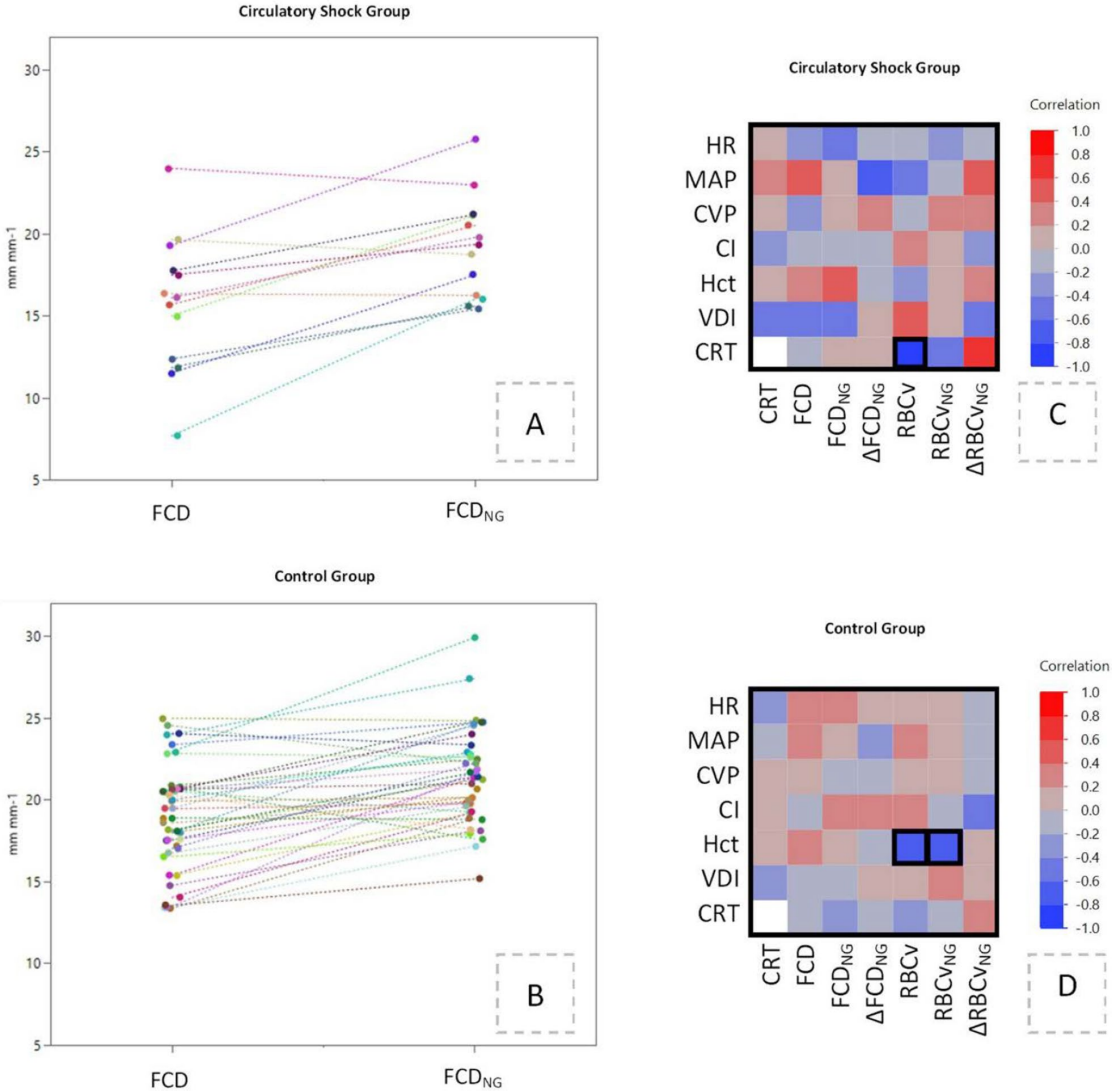
Individual measurements of FCD and FCD_NG_ in patients with circulatory shock (**A**) and controls (**B**), demonstrating a consistent nitroglycerin response in both groups. **C**, **D** Shows linear correlation coefficients (color scale) between macrohemodynamic variables on the vertical axis and microhemodynamic variables on the horizontal axis. An association between sublingual RBCv and peripheral capillary refill time was shown in the circulatory shock group, and a negative association between RBCv and its nitroglycerin response, and systemic hematocrit was found in the control group. Correlation pairs with *r* ≥ 0.6 or *r* ≤ − 0.6, and *p* ≤ 0.05 after Bonferroni’s correction are encircled with a solid black line in both panels. *CRT* capillary refill time, *VDI* vasopressor dependency index, *HR* heart rate, *MAP* mean arterial pressure, *CVP* central venous pressure, *Hct* systemic hematocrit, *FCD* functional capillary density, *RBCv* red blood cell velocity, *FCD*_*NG*_ maximal recruitable functional capillary density, *RBCv*_*NG*_ maximal recruitable red blood cell velocity, *∆FCD*_*NG*_ functional capillary density reserve capacity, *∆RBCv*_*NG*_ functional red blood cell velocity reserve capacity

The sublingual nitroglycerin challenge increased FCD by 22% in patients with circulatory shock (within-group *p* = 0.03) and 13% in patients of the control group (within-group *p* < 0.001, Figs. 2A and 3A). FCD_NG_ was 10% lower in patients with circulatory shock as compared to controls (between-group *p* = 0.03, Figs. 2A and 3B), but ΔFCD_NG_ remained similar between the two groups (*p* = 0.19, Fig. 2B). Circulatory shock patients reached FCD_NG_ similar to FCD in patients without shock (Fig. 2A). RBCv_NG_ was similar to RBCv in both groups (within-group *p* > 0.05, Fig. 2C), with similar RBCv_NG_ and ∆RBCv_NG_ in both groups (between-group *p* = 0.97 and *p* = 0.25, Fig. 2B, D).

### Outcome and relationship between macro- and microhemodynamic variables

Microcirculatory variables including nitroglycerin response were similar in 28-day survivors and non-survivors in both groups, with FCD and FCD_NG_ of 18.0 ± 3.7 and 21.0 ± 3.1 in survivors, and 17.0 ± 3.6 and 20.0 ± 2.8 in non-survivors (*p* = 0.29 and *p* = 0.08, respectively). No clinically relevant association (*r* ≥ 0.6, *p* < 0.05) was found between FCD and its nitroglycerin response, and arterial or central venous blood pressure, cardiac output, heart rate, vasopressor dependency index, hematocrit, or capillary refill time (Fig. 3C, D, Supplementary Table S2). In the circulatory shock group, RBCv measured in the sublingual microcirculation was associated with capillary refill time measured on the fingernail (*r* = − 0.81, *p* < 0.001), while in the control group RBCv and its nitroglycerin response were negatively correlated to systemic hematocrit (*r* = − 0.65, *p* < 0.0001 for both variables). In the 13 patients with sepsis, with and without shock, 28-day mortality was associated with lower FCD and FCD_NG_ (*p* = 0.018 and *p* = 0.012, respectively). In these patients, FCD was negatively associated with the vasopressor dependency index (*r* = 0.71, *p* < 0.001).

## Discussion

In severely ill patients with circulatory shock, a lower FCD and FCD_NG_, but not RBCv and RBCv_NG_, was found after initial resuscitation, as compared to patients without circulatory shock. The response to a topical nitroglycerin stimulus of FCD and RBCv was similar in patients with circulatory shock as compared to patients without circulatory shock, and FCD_NG_ in patients with circulatory shock was similar to that in native FCD in patients without circulatory shock. No association between nitroglycerin response and blood pressure or cardiac output was found regardless of the presence of circulatory shock.

### Circulatory shock is associated with lower native microcirculatory diffusion capacity

Previous studies have shown impaired microcirculatory function in septic and cardiogenic shock [2, 6, 27]. These findings were based on the measurement of total vessel density combined with the subjective visual flow characteristics of red blood cells within the capillaries to enable the calculation of the perfused vessel density, as described in the first consensus on the evaluation of the microcirculation [28]. They mainly revealed an association between shock and the subjectively graded microvascular flow index (MFI) or proportion of perfused vessels (PPV) derived from vessel-by-vessel assessment of the former. In the present study, the data was collected in a severely ill cohort in both the shock and control group, as reflected by high SAPS and SOFA scores and 28-day mortality of 46 and 30%, respectively. Our data suggest, by quantifying the variables associated with diffusion and convection capacity of the oxygen carriers in the tissue, as suggested by the second consensus on the evaluation of the microcirculation [21, 28, 29], that the main variable affected by circulatory shock of any cause is FCD as opposed to RBCv, implying the microcirculatory diffusion capacity may be a more sensitive indicator of the presence of circulatory shock than changes in the convection capacity. Previous studies have further described an association between the presence of circulatory shock and abnormalities of the skin microcirculation such as capillary refill time and the mottling score [16, 17]. It has, however, remained unclear how these variables might be associated with the functional state of the sublingual microcirculation, which is regulated more similarly to the visceral microcirculation [30]. In the present study, sublingual RBCv was found to be associated with capillary refill time assessed in the peripheral microcirculation in the presence of circulatory shock. The same was not found to be true for FCD or the nitroglycerin response. In patients with circulatory shock, the peripheral capillary refill time may thus be more representative of the sublingual microcirculatory convection capacity as opposed to the sublingual microcirculatory diffusion capacity, meaning that FCD is a more sensitive indicator of the presence of circulatory shock than either RBCv or the capillary refill time.

### Microcirculatory nitroglycerin response is preserved in patients with circulatory shock

The nitroglycerin response of the sublingual microcirculation has previously been suggested as a method to quantify the microcirculatory reserve capacity to adapt to deficiencies in oxygen availability in the tissue in healthy volunteers exposed to severe hypobaric hypoxia [22]. Correspondingly, data previously collected in mechanically ventilated patients suffering from COVID-19 have indicated that a similar mechanism to increase oxygen extraction capacity at the level of the tissue was preserved even in these critically ill patients [31–33]. These results contrasted earlier findings in patients suffering from septic and cardiogenic shock who presented with deteriorated microcirculatory function secondary to circulatory shock [2, 6]. In these patients, red blood cell flow in the sublingual microcirculation was increased by the topical application of acetylcholine, which stimulates the endothelial cells to release nitric oxide and nitroglycerin [2, 6, 23]. These studies, however, did not examine how the response described in circulatory shock compared to patients without circulatory shock. Nevertheless, these findings supported the hypothesis that the presence of a capillary reserve capacity would be able to be resuscitated by procedures to augment the oxygen extraction capacity of the tissues following circulatory shock. The present study showed that the sublingual microcirculatory response to nitroglycerin with respect to FCD was comparable regardless of the presence of circulatory shock indicating the preservation of a window to recruit the microcirculatory reserve following circulatory shock, and even in the presence of nitroglycerin as a direct nitric oxide donor as opposed to acetylcholine and thus bypassing the need to provision nitric oxide by the endothelial cells. This finding suggests that despite the inadequate oxygen availability in the tissue characteristic for circulatory shock, the regulatory mechanisms to increase oxygen extraction capacity in the tissue previously described in healthy volunteers seem not to occur in the presence of circulatory shock. A suggested failure of these underlying intrinsic compensatory mechanisms of the microcirculation in response to shock is further supported by the lower maximal recruitable FCD after the nitroglycerin challenge as compared to patients without circulatory shock. A contributing factor to this effect could be the presence of fluid overload related to volume resuscitation [34] as suggested by a higher central venous pressure—which effectively represents the outlet pressure of the systemic microcirculation—and/or cardiac congestion in patients suffering from circulatory shock, and also reduction of viscosity of blood possibly negatively affecting sheer stress related autoregulatory mechanisms. These latter explanations would be consistent with the negative nitroglycerin response with respect to RBCv and compatible with previous association of the microcirculatory convection capacity with microcirculatory tamponade caused by an increase in capillary outlet pressure [9, 35]. Another study, examining patients with cardiogenic shock after cardiac surgery, also reported a trend toward higher central venous pressure in non-responders to a nitroglycerin stimulus [23]. These data confirm the present finding of a preserved nitroglycerin response in the sublingual microcirculation in circulatory shock, also in the presence of circulatory failure after cardiac surgery. Furthermore, a reduced capillary density in patients undergoing major cardiac surgery has previously been associated with a highly positive fluid balance in the first 7 days after cardiac surgery [36, 37]. Finally, the association between persistent microcirculatory dysfunction after initial resuscitation and mortality, which has also been shown in previous studies mainly for patients suffering from septic shock [38], was confirmed in our data by both lower FCD and FCD_NG_ in 28-day non-survivors as compared to survivors suffering from sepsis. Larger, longitudinal studies are warranted to differentiate the effects of different etiologies of circulatory shock and resuscitation measures on the microcirculatory hemodynamic variables and nitroglycerin response, and on outcome [39].

### The nitroglycerin response in the sublingual microcirculation may identify a tissue perfusion target in circulatory shock

The main treatment goal in circulatory shock is the support of rapid restitution of tissue perfusion and oxygenation. Consistently, the restoration of inadequate tissue perfusion should be a main target for resuscitation. Our primary finding in this study, however, is that despite the application of standard resuscitation procedures, the FCD in resuscitated circulatory shock patients remains lower than in control patients. A second main finding is that a sublingual challenge by topical application of nitroglycerin identifies the amount of FCD still in need of recruitment to arrive at baseline FCD as found in control patients. This was shown in our study by the observation that the level of increased FCD in the resuscitated circulatory shock patients induced by the nitroglycerin challenge corresponds to baseline FCD in the control patients. The significance of these findings is that it may open the way to microcirculatory tissue perfusion guided resuscitation where a nitroglycerin challenge can quantitatively identify and define the tissue perfusion target needed to achieve adequate tissue perfusion.

## Limitations

The present study has several limitations. First, it is observational in nature and was conducted without obtaining longitudinal measurements. The in vivo measurement of nitroglycerin response in the sublingual microcirculation could in future studies be used in a larger patient population and to describe longitudinal changes throughout ICU treatment. Further, due to the lack of previous data regarding the microcirculatory nitroglycerin response in patients with circulatory shock, the number of included patients was based on differences in FCD induced by circulatory shock. The study was not powered to perform a sub-analysis of patients with specific forms of circulatory shock, however the use of a common definition of circulatory shock, including septic and cardiogenic shock as previously described [40], allowed the systematic examination of common properties of circulatory shock. The results of this preliminary study serve as a validation of a physiological description of the mechanisms of circulatory shock in critically ill patients and as the basis for the inclusion of specific types of circulatory shock in larger cohorts in the future. Finally, the topical application of nitroglycerin, as performed in the present study, does not allow the prediction of the effect of systemic resuscitation measures, including the systemic application of vasodilators such as nitroglycerin as described previously [41, 42], but rather provides a tool for the assessment of the functional state of the microcirculation.

## Conclusion

In the present study, critically ill patients suffering from circulatory shock were found to have a lower sublingual FCD as compared to patients without shock. The preserved response to a topical nitroglycerin stimulus after initial resuscitation, alongside the lower FCD_NG_, furthermore suggests an inability of the intrinsic regulation mechanisms to increase the microcirculatory oxygen extraction capacity associated with circulatory shock and thereby identifies a potential resuscitation target. These differences in microcirculatory hemodynamic function between patients with and without circulatory shock were neither reflected in blood pressure nor cardiac index, highlighting the potential of guiding resuscitation in these patients according to the measurement of tissue perfusion and tissue oxygen availability in future studies.

## Supplementary Information

Supplementary Material 1.

## Data Availability

The datasets generated and analyzed during the current study are not publicly available due to sensible personal clinical data, but are available from the corresponding author upon reasonable request.
