# Supplementary material for: Nitroglycerin challenge identifies microcirculatory target for improved resuscitation in patients with circulatory shock
Source: Intensive Care Med Exp. 2024 Sep 2;12:76. doi: 10.1186/s40635-024-00662-3 (PMC11369126; doi:10.1186/s40635-024-00662-3)
Supplement: Supplementary file 1 — Supplementary Material 1. [file 40635_2024_662_MOESM1_ESM.pdf]

Supplementary Information

Nitroglycerin challenge identifies microcirculatory target for improved resuscitation in patients with circulatory shock

Massimiliano BERTACCHI, Pedro D. WENDEL-GARCIA, Anisa HANA,

Can INCE, Marco MAGGIORINI, Matthias P. HILTY

Supplementary Table S1: Estimates for microcirculatory variables of patients included in the control group and the circulatory shock group. 2

Supplementary Table S2: Linear correlation between microcirculatory and hemodynamic variables in patients with and without circulatory shock.................... 3

Supplementary Table S3. Microcirculatory hemodynamics in patients with and without veno-arterial extracorporeal membrane oxygenation................................. 4

Supplementary Table S1: Estimates for microcirculatory variables of patients included in the control group and the circulatory shock group.

Model estimates represented by the mean difference, 95% confidence intervals and p values as obtained via linear mixed model analysis with circulatory shock status entered as fixed effects and intercepts for subjects and per-subject random slopes representing the effect on the dependent variables entered as random effects. FCD, functional capillary density; RBCv, red blood cell velocity; FCD_{NG}, maximal recruitable functional capillary density; $RBCv_{NG}$, maximal recruitable red blood cell velocity; ΔFCD_{NG}, functional capillary density reserve capacity; $\Delta RBCv_{NG}$, functional red blood cell velocity reserve capacity.

	**Control Group**<br>**n = 41**	**Circulatory Shock Group**<br>**n = 13**	**p value**
**Baseline microcirculation**			
FCD [mm mm^{-2}]	18.9 (17.0 - 20.6)	16.1 (12.4 - 17.7)	**<0.01**
RBCv [µm s^{-1}]	345 (322 - 381)	348 (329 - 409)	0.48
**Maximal recruitable capacity**			
FCD_{NG} [mm mm^{-2}]	21.3 (19.3 - 22.8)	19.3 (16.2 - 21.1)	**0.03**
$RBCv_{NG}$ [µm s^{-1}]	351 (335 - 369)	353 (331 - 372)	0.97
**Microcirculatory reserve capacity**			
ΔFCD_{NG} [mm mm^{-2}]	2.4 (0.3 - 3.9)	3.7 (1.9 – 6.0)	0.19
$\Delta RBCv_{NG}$ [µm s^{-1}]	11 (-8 - 23)	-4 (-37 - 30)	0.25

Supplementary Table S2: Linear correlation between microcirculatory and hemodynamic variables in patients with and without circulatory shock.

Correlation pairs with $r \geq 0.6$ or $r \leq -0.6$, and $p \leq 0.05$ after Bonferroni's correction are highlighted in blue and red, respectively. CRT, capillary refill time; VDI, vasopressor dependency index; HR, heart rate; MAP, mean arterial pressure; CVP, central venous pressure; Hct, systemic hematocrit; FCD, functional capillary density; RBCv, red blood cell velocity; FCD_{NG}, maximal recruitable functional capillary density; $RBCv_{NG}$, maximal recruitable red blood cell velocity; ΔFCD_{NG}, functional capillary density reserve capacity; $\Delta RBCv_{NG}$, functional red blood cell velocity reserve capacity.

All patients

Correlation coefficients	CRT	FCD	FCD_{NG}	ΔFCD_{NG}	RBCv	$RBCv_{NG}$	$\Delta RBCv_{NG}$	p values with Bonferroni correction	CRT	FCD	FCD_{NG}	ΔFCD_{NG}	RBCv	$RBCv_{NG}$	$\Delta RBCv_{NG}$
HR	0.09	-0.07	0.01	0.11	0.12	0.01	-0.16	HR	1.00	1.00	1.00	1.00	1.00	1.00	1.00
MAP	0.02	0.49	0.24	-0.4	-0.04	0.09	0.14	MAP	1.00	**<0.01**	1.00	0.14	1.00	1.00	1.00
CVP	0.18	-0.13	-0.1	0.07	0.07	0.1	0.01	CVP	1.00	1.00	1.00	1.00	1.00	1.00	1.00
CI	-0.13	-0.13	0.01	0.17	0.27	-0.03	-0.41	CI	1.00	1.00	1.00	1.00	1.00	1.00	1.00
Hct	0.09	0.25	0.22	-0.09	-0.5	-0.48	0.2	Hct	1.00	1.00	1.00	1.00	**<0.01**	0.01	1.00
VDI	-0.07	-0.43	-0.36	0.18	0.33	0.14	-0.31	VDI	1.00	**0.05**	0.38	1.00	0.96	1.00	0.96
CRT	-	-0.2	-0.19	0.08	-0.5	-0.29	0.39	CRT	-	1.00	1.00	1.00	**<0.01**	1.00	0.24

Circulatory Shock Group

Correlation coefficients	CRT	FCD	FCD_{NG}	ΔFCD_{NG}	RBCv	$RBCv_{NG}$	$\Delta RBCv_{NG}$	p values with Bonferroni correction	CRT	FCD	FCD_{NG}	ΔFCD_{NG}	RBCv	$RBCv_{NG}$	$\Delta RBCv_{NG}$
HR	0.16	-0.27	-0.41	-0.05	-0.13	-0.26	-0.02	HR	1.00	1.00	1.00	1.00	1.00	1.00	1.00
MAP	0.38	0.55	0.17	-0.61	-0.45	-0.08	0.57	MAP	1.00	1.00	1.00	1.00	1.00	1.00	1.00
CVP	0.04	-0.22	0.04	0.36	-0.02	0.29	0.26	CVP	1.00	1.00	1.00	1.00	1.00	1.00	1.00
CI	-0.28	-0.17	-0.19	-0.03	0.25	0.08	-0.26	CI	1.00	1.00	1.00	1.00	1.00	1.00	1.00
Hct	0.07	0.33	0.41	-0.04	-0.23	0.08	0.38	Hct	1.00	1.00	1.00	1.00	1.00	1.00	1.00
VDI	-0.44	-0.5	-0.49	0.19	0.45	0.18	-0.48	VDI	1.00	1.00	1.00	1.00	1.00	1.00	1.00
CRT	-	-0.01	0.12	0.14	**-0.82**	-0.53	0.71	CRT	-	1.00	1.00	1.00	**0.03**	1.00	0.34

Control Group

Correlation coefficients	CRT	FCD	FCD_{NG}	ΔFCD_{NG}	RBCv	$RBCv_{NG}$	$\Delta RBCv_{NG}$	p values with Bonferroni correction	CRT	FCD	FCD_{NG}	ΔFCD_{NG}	RBCv	$RBCv_{NG}$	$\Delta RBCv_{NG}$
HR	-0.23	0.24	0.33	0.05	0.18	0.07	-0.15	HR	1.00	1.00	1.00	1.00	1.00	1.00	1.00
MAP	-0.06	0.4	0.19	-0.28	0.24	0.16	-0.17	MAP	1.00	0.48	1.00	1.00	1.00	1.00	1.00
CVP	0.13	0.09	-0.03	-0.14	0.09	0.02	-0.1	CVP	1.00	1.00	1.00	1.00	1.00	1.00	1.00
CI	0.02	-0.09	0.21	0.30	0.31	-0.09	-0.59	CI	1.00	1.00	1.00	1.00	1.00	1.00	0.96
Hct	0.18	0.21	0.14	-0.09	**-0.65**	**-0.65**	0.11	Hct	1.00	1.00	1.00	1.00	**<0.01**	**<0.01**	1.00
VDI	-0.23	-0.05	-0.05	0.01	0.17	0.23	0.06	VDI	1.00	1.00	1.00	1.00	1.00	1.00	1.00
CRT	-	-0.15	-0.24	-0.06	-0.33	-0.18	0.21	CRT	-	1.00	1.00	1.00	1.00	1.00	1.00

Supplementary Table S3. Microcirculatory hemodynamics in patients with and without veno-arterial extracorporeal membrane oxygenation.

va-ECMO, veno-arterial extracorporeal membrane oxygenation; FCD, functional capillary density; RBCv, red blood cell velocity; FCDNG, maximal recruitable functional capillary density; RBCvNG, maximal recruitable red blood cell velocity; ΔFCDNG, functional capillary density reserve; ΔRBCvNG, red blood cell velocity reserve.

	**Without extracorporeal membrane oxygenation**	**With veno-arterial extracorporeal membrane oxygenation**
**Circulatory Shock Group**	**n = 10**	**n = 3**
FCD [mm mm^{-2}]	16.1 (14.1 - 18.0)	14.7 (-6.2 - 35.5)
FCD_{NG} [mm mm^{-2}]	19.6 (17.5 - 21.7)	18.1 (7.7 - 28.6)
ΔFCD_{NG} [mm mm^{-2}]	3.5 (1.7 - 5.4)	3.5 (-8.2– 15.1)
RBCv [µm s^{-1}]	340 (300 - 379)	413 (274 - 552)
$RBCv_{NG}$ [µm s^{-1}]	349 (321 - 376)	364 (307 - 420)
$\Delta RBCv_{NG}$ [µm s^{-1}]	9 (-19 - 37)	-50 (-133 - 34)
**Control Group**	**n = 35**	**n = 6**
FCD [mm mm^{-2}]	19.0 (17.9 - 20.1)	18.2 (15.4 - 21.0)
FCD_{NG} [mm mm^{-2}]	21.6 (20.3 - 22.5)	19.6 (15.7 - 23.5)
ΔFCD_{NG} [mm mm^{-2}]	2.6 (1.6 - 3.5)	1.3 (-1.0 - 3.5)
RBCv [µm s^{-1}]	340 (328 - 352)	385 (371 - 399)
$RBCv_{NG}$ [µm s^{-1}]	350 (339 - 361)	361 (337 - 686)
$\Delta RBCv_{NG}$ [µm s^{-1}]	11 (3 - 18)	-21 (-53 - 11)
